# The first case of hand infection caused by *Dermabacter jinjuensis* in a symmetrical peripheral gangrene patient

**DOI:** 10.1016/j.amsu.2018.10.008

**Published:** 2018-10-16

**Authors:** Seong Hee Cho, Jin Sung Park, Woo-Kon Lee, Min-Kyoung Shin, Myunghwan Jung, Kyeong Min Lee, Kyu Jam Hwang, Dong Kyu Moon

**Affiliations:** a Department of Orthopaedic Surgery and Institute of Health Sciences, Gyeongsang National University School of Medicine and Gyeongsang National University Hospital, Jinju, Republic of Korea; b Department of Orthopaedic Surgery, Seoul National University College of Medicine, Seoul National University Bundang Hospital, Seongnam, Republic of Korea; c Pathogen Resource TF, Center for Infectious Diseases, Korea National Institute of Health, Korea Center for Disease Control and Prevention, Cheongju, Republic of Korea; d Department of Microbiology, School of Medicine, Gyeongsang National University, Jinju, South Korea

**Keywords:** *Dermabacter jinjuensis*, *Dermabacter*, Hand infection, Symmetrical peripheral gangrene, Case report

## Abstract

**Introduction:**

Strains of the genus *Dermabacter* is a recently established species, recognized as relatively rare opportunistic human pathogen, and is infrequently isolated from clinical specimens, including blood cultures, abscesses, wounds, bone, eye, and skin.

**Presentation of case:**

We present a 78-year old female with chronic symmetrical peripheral gangrene and hand infection. The patient underwent surgical debridement with amputation on gangrene with infection of both fingers. At 2 weeks postoperatively, pus discharge was newly observed and the patient underwent reoperation. In the subsequent reinfection, unknown organism has been repeatedly identified, may be the most likely causative agent. On the basis of phenotypic and genotypic distinctness and DNA–DNA hybridization results, new strain should be placed in the genus *Dermabacter* as representing a novel species, for which the name *Dermabacter jinjuensis* sp. nov. is proposed.

**Discussion:**

We judged the novel species as the causative bacteria. Because of, a novel species called *D. jinjuensis* was repeatedly identified more than common bacteria. It can be considered as a postoperative nosocomial infection or opportunistic infection. It is not clear how the infection of *D. jinjensis* occurred.

**Conclusion:**

This is the first reported case of a human *D. jinjuensis* infection. We were able to treat patients without any complications by operative treatment and administering appropriate antimicrobial agents according to antibiotics susceptibility test.

## Highlights

• Strain of the genus *Dermabacter* is a recently established species.
• Strain of the genus *Dermabacter* recognized as relatively rare opportunistic human pathogen.
• *Dermabacter jinjuensis* is newly described *Dermabacter* species.
• *Dermabacter jinjuensis* can be human pathogen.

## 1 Introduction

Strains of the genus *Dermabacter* were first isolated from human skin and assigned to a novel species of this new genus, *Dermabacter hominis* [1]. *D. hominis* is a recently established species, recognized as relatively rare opportunistic human pathogen, and is infrequently isolated from clinical specimens, including blood cultures, abscesses, wounds, bone, eye, and skin [[2], [3], [4], [5], [6], [7]]. Recently, *Dermabacter vaginalis* first isolated from vaginal discharge in women has been reported, and *Dermabacter indicies* isolated from the wounds after being bitten by fish have been reported [8,9]. However, it is not known whether these bacteria are clinically significant yet. We have recently published a clinically newly discovered microbiology of *Dermabacter jinjuensis* and have been recognized as a new strain [10]. However, we did not report how the bacterium was clinically discovered and how it was treated. Therefore, we report a clinical case of hand infection caused by *D. jinjuensis*. The present case is reported in line with the SCARE criteria [11].

## 2 Case report

**Fig. 1:**
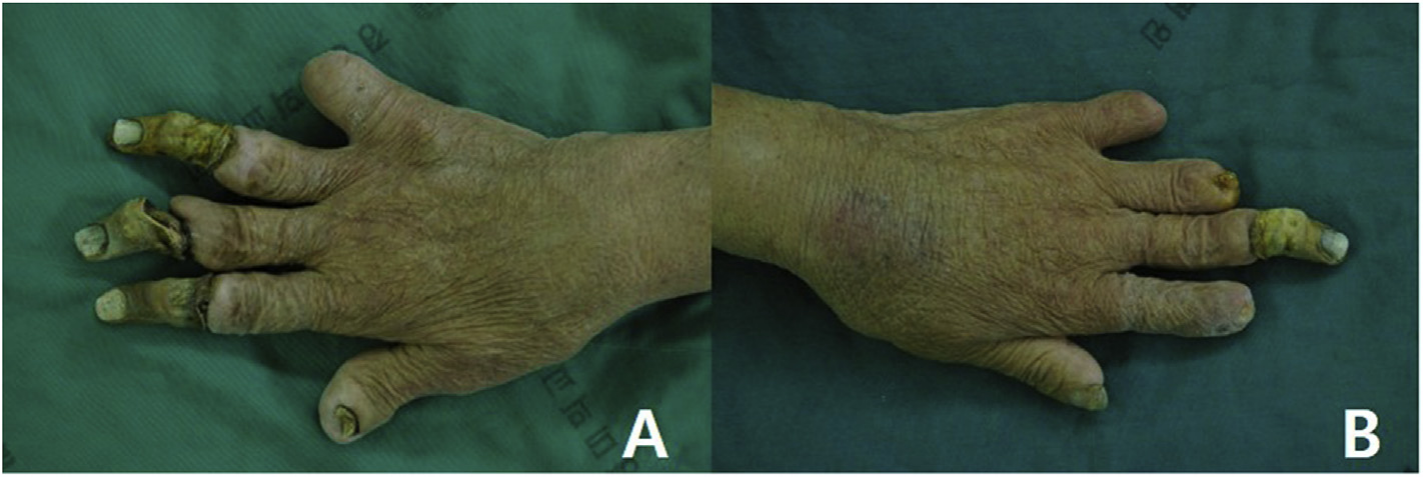
A, Preoperative gross picture of right hand demonstrate 2nd, 3rd, and 4th fingers gangrene in the distal part of the proximal interphalangeal (PIP) joint, and the 5th finger spontaneous amputation at the PIP joint level. B, Preoperative gross picture of left hand demonstrate 2nd, 4th, and 5th finger spontaneous amputation and draining sinus on amputated stump.

A 78 - year - old female visited the outpatient clinic with bilateral multiple finger dry gangrene and a pus discharge. On physical examination, the right 2nd, 3rd, and 4th fingers showed dry gangrene in the distal part of the proximal interphalangeal (PIP) joint, and the 5th finger was spontaneously amputated at the PIP joint level. The left 3rd finger showed dry gangrene in the distal part of PIP joint, and the 2nd, 4th, and 5th finger were spontaneously amputated at the middle phalanx level. Left 2nd and 4th finger and right 5th finger presented with draining sinus on amputated stump (Fig. 1).

In social history, she lived in urban area (Sacheon city, Gyeongsangnam-do, Republic of Korea). She was housekeeper. In past medical history, she was taking antihypertensive drugs and oral steroids for a year because of hypertension and adrenal insufficiency. One year ago, she received ICU treatment for toxic shock syndrome due to left lower extremity cellulitis. At that time, symmetrical peripheral gangrene (SPG) occurred in both fingers as a complication of high dose dopamine administration. At that time, the patient needed multiple finger amputation for SPG. However, the patient refused operative treatment at that time and the management was delayed. Three months ago, pus discharge was observed in the gangrene area. The patient was dressed in the local hospital but visited for amputation without improvement of symptoms. Preoperative laboratory findings showed WBC count 5680/mm^3^, ESR 49mm/hr, CRP 1.9mg/L.

**Fig. 2:**
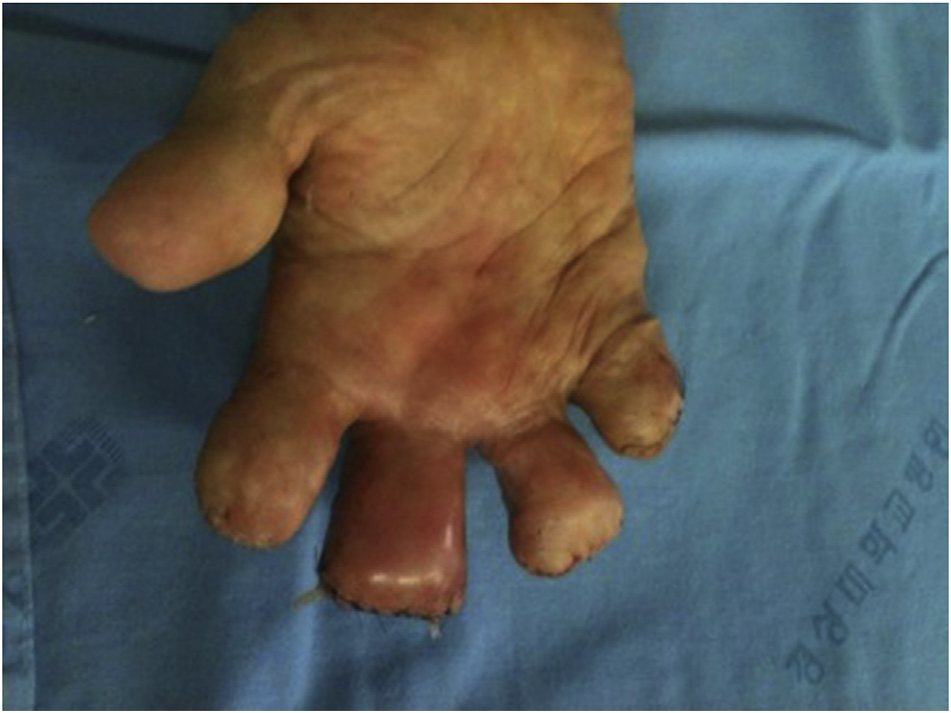
Gross picture of right hand at 2 weeks after first operation demonstrate swelling, erythematous change and pus drainage of 3rd finger amputation stump.

The patient underwent surgical debridement with amputation on gangrene with infection and performed amputated stump revision. Preoperative and intraoperative Gram stain showed Gram-negative bacilli. After microbial cultivation, Vitek 2 system (bioMérieux Inc., Durham, NC, USA) was used for bacterial identification. *Pseudomonas aeruginosa* was identified in aerobic culture, but nothing was shown in anaerobic culture. According to antibiotics susceptibility test, intravenous ciprofloxacin was administered for infection management. However, on 10th postoperative day, right 3rd finger amputation stump area presented with swelling and redness. At 2 weeks postoperatively, pus discharge was newly observed and infection sign spread to the proximal area (Fig. 2). Laboratory findings showed WBC count 7480/mm^3^, ESR 39mm/hr, CRP 40.9mg/L. We diagnosed with pyogenic tenosynovitis. We performed infectious tissue debridement, feeding tube insertion for irrigation, and stump revision.

**Fig. 3:**
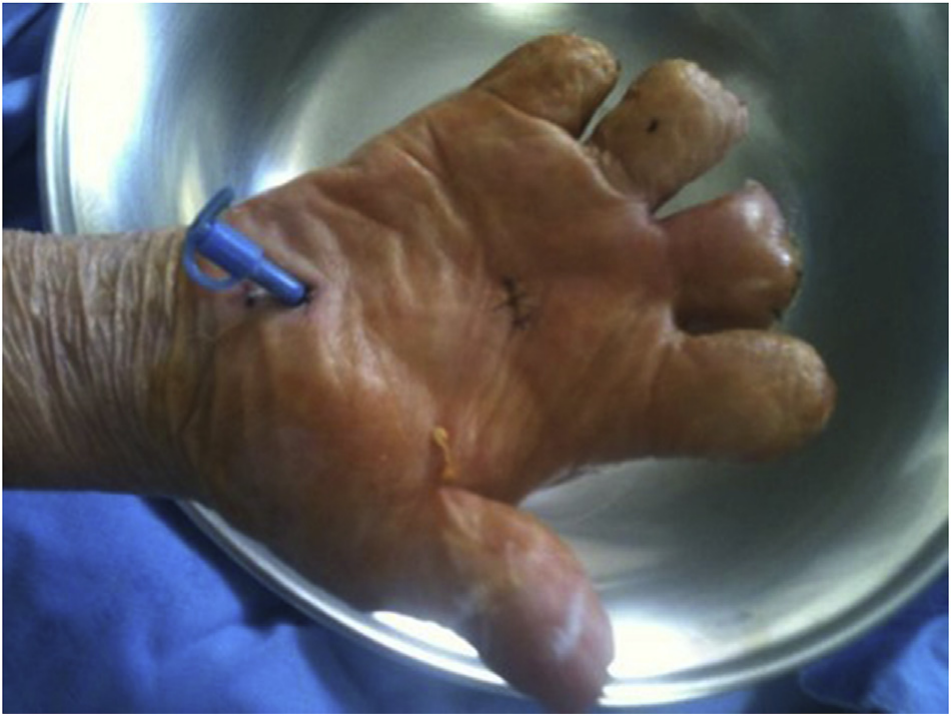
Gross picture of right hand at 7 days after second operation demonstrate mild swelling, erythematous change of 3rd finger amputation stump.

On the first day of pus discharge, we performed Gram staining of the open pus and microbial cultivation. Gram-positive coryneform and Gram-negative bacilli were observed simultaneously in Gram stain. On 6th postoperative day, viridans streptococci were identified in aerobic culture and *Bacteroides stercoris* was identified in both aerobic and anaerobic culture using the equipment described above. On a day before reoperation, Gram staining and culture were performed again. In Gram stain, Gram-positive coryneform and Gram-negative bacilli were observed this time as before. On 6th postoperative day, *Dermacoccus nishnomiyaensis* and *Kytococcus sedentarius* were identified in aerobic culture. According to antibiotics susceptibility test, intravenous penicillin-G administration was added. Gram stain during reoperation showed Gram-positive coryneform and Gram-negative bacilli persistently. We performed two intraoperative bacteriological examination. On 10th postoperative day, *B. stercoris* was identified in both aerobic and anaerobic culture. In the subsequent reinfection, *B. stercoris* has been repeatedly identified, may be the most likely causative agent. The results of antibiotics susceptibility test were as shown in Table 1. However, other bacteria may also be pathogenic organisms. Therefore, according to antibiotics susceptibility test, piperacillin/tazobactam, effective to all identified bacteria, was administered intravenously for 16 days. CRP was normalized 10 days after reoperation. After the operation, irrigation was performed twice a day through a feeding tube for 18 days (Fig. 3). Oral antibiotics have been substituted (amoxicillin/clavulanic acid) for 14 days. The patient recovered uneventfully. There has been no reinfection or complication in the final follow-up of the patient for 1 years after the operation.

**Table 1:** Antibiotics susceptibility test of dermabacter jinjuensis.

Antibiotics	MIC	Susceptibility
Penicillin	<1㎍/mL	S
Imipenem	<4㎍/mL	S
Vancomycin	<2㎍/mL	S
Ciprofloxacin	>4㎍/mL	R

However, the *Bacteroides* species were generally known to be a gram-negative, obligate anaerobic bacterium that cannot grow in aerobic condition. In this case, we have been questioning the proliferation of this bacteria in both aerobic and anaerobic conditions. Therefore, we performed additional studies on the assumption that they could be bacteria other than the *Bacteroides* species. New strain was considered as prepresentating a nevel species according to its initial identification by matrix-assisted laser desorption/ionization-time-of-flight MS. Phylogenetic analysis based on 16S rRNA gene sequences revealed that new strain belonged to the genus *Dermabacter* and was closely related to *D. hominis* DSM 7083^T^ (=ATCC 49369^T^) (98.34%), type strains of *Helcobacillus massiliensis* (96.67%). New strain was a Gram-stain-positive, catalase-positive, non-motile, coryneform-like coccobacillus bacterium. Optimal growth was observed at 30–40 °C and pH 7. New strain was able to grow anaerobically and exhibited a tolerance range for NaCl of 0–6% (w/v). Menaquinones MK-8, MK-7 and MK-9 were the major quinones. Major polar lipids were phosphatidylethanolamine, phosphatidylcholine, glycolipid, two unknown lipids and an unknown phospholipid. The G + C content of new strain was 63.16 mol% and the major fatty acids were anteiso-C_17:0_, anteiso-C_15:0_, iso-C_16:0_ and iso-C_15:0_. The mean level of DNA–DNA relatedness between new strain and *D. hominis* ATCC 49369^T^ was 49 ± 1.6%, indicating that new strain represents a novel species.

On the basis of phenotypic and genotypic distinctness and DNA–DNA hybridization results, new strain should be placed in the genus *Dermabacter* as representing a novel species, for which the name *Dermabacter jinjuensis* sp. nov. is proposed and detailed microbiological information was described in the previous paper [10].

## 3 Discussion

*Staphylococcus aureus* and beta-hemolytic streptococci are the most common bacterial culprits of acute hand infections [12]. Although the infectious organism is most often a gram-positive bacteria, gram-negative, mycobacterial, or fungal organisms can also cause chronic infections. Over half of all the chronic infections are polymicrobial [13]. In this case, the spontaneous amputee of the bilateral hand and chronic infection of the SPG site were observed physically at the time of admission. *P. aerusinosa* was identified in bacteriological examination and antibiotics were administered according to the results of appropriate antibiotics sensitivity. However, postoperative acute infection was observed and polymicrobial infection was identified in bacteriological examination, but a novel species called *D. jinjuensis* was repeatedly identified more than common bacteria. Therefore, we judged the novel species as the causative bacteria.

Dopamine is frequently used for the treatment of patients with septic shock due to its effects on enhancing myocardial contractility and vasoconstriction [14]. However, vasoconstriction can cause side effects including skin ischemia and peripheral circulatory disturbance in the extremities [15,16]. The event of ischemia progressing to result in SPG is rare. However, if it occurs, the affected area should be amputated. In this case, the patient refused amputation when SPG occurred and the significant amount of time has passed until emergence of infection sign. This suggests that a chronic infection has occurred during that period.

*D. hominis* as one of the human skin flora, was first considered to be non-pathogenic [1]. Nevertheless, recently, *D. hominis* has been reported as an opportunistic human pathogen [4,6,7]. It is not clear how the infection of *D. jinjensis* occurred. *D. jinjuensis* was first identified in the obtained specimen about 2 weeks after the first operation. Therefore, it can be considered as a postoperative nosocomial infection. The basis for the consideration is that the patient was in the immune compromised state because she was old and was undergoing steroid therapy for a long time due to adrenal insufficiency. Furthermore, the role of the barrier of normal skin was disrupted due to chronic SPG. Therefore, there is a possibility that the *D. jinjuenesis* has already been contaminated *in situ*. Chronic infection due to *P. aerusinosa* and *D. jinjuenesis* are present in contamination state. It is possible that the use of antibiotics after initial surgery may cause opportunistic infection of *D. jinjuensis* due to decrease in the number of other bacteria including *P. aerusinosa*. In this study, *D. jinjuensis* was resistant to ciprofloxacin in the antibiotics susceptibility test. Gómez-Garcės et al. [3] reported that the use of broad spectrum antibiotics could opportunistically develop the virulence of *D. hominis* in patients with severe underlying disease and reduction of defensive capacity. However, since it is difficult to generalize it as opportunistic infection of D. jinjuensis, additional research is needed.

Antibiotics which *D. hominis* is susceptible to are known to be tetracyclines, most aminoglycosides, carbapenems, macrolides, lincosamides, glycopeptides, rifampin, and linezolid. In contrast, it is known to be resistant to pipemidic acid, sulfamethoxazole, and cotrimoxazole [17,18]. However, clinical isolate of *D. hominis* differs in antibiotics susceptibility according to the study [4,6,7,19]. In this case, antibiotics susceptibility test was performed in all bacteria, and intravenous piperacillin/tazobactam, which all identified bacteria are susceptible to, was used. In addition, oral amoxicillin/clavulanic acid was used for 2 weeks after disappearance of clinical infection sign.

In previous reports, the patient died from infection by *D. hominis*, but all cases occurred in acute bacteremia. No long term complication after recovery was described in terms of localized infection. Long term complications did not occur in this study due to infection by *D. jinjuensis* so far. This study suggests that the infection was localized only in the hand. However, further study will be needed in relation to complication.

## 4 Conclusion

In conclusion, to the best of our knowledge, this is the first reported case of a human *D. jinjuensis* infection. We were able to treat patients without any complications by operative treatment and administering appropriate antimicrobial agents according to antibiotics susceptibility test.

## Conflicts of interest

All of the Authors declare that they have no conflict of interest either personally or with any of their relatives.

## Sources of funding

All authors declare that they did not receive any source of funding by any mean to run this case report. They wrote this paper and they edit it on their own fund.

## Ethical approval

The retrospective case report is exempt from ethical approval in our institution.

## Research registration number (UIN)

This report don not need registration.

## Trial registry number

This report don not need registration.

## Author contribution

**Dong Kyu Moon**: is the corresponding author. He contributed in study design, data collection and analysis, writing paper, and reviewing literature.

**Sung hee Cho**: Study design, data analysis, writing the paper, and reviewing literature.

**Jin Sung Park and Woo Kon Lee**: Study design, data analysis and reviewing literature.

**Min Kyoung Shin, Myunghwan Jung, Kyeong Min Lee and Kyu Jam Hwang**: Study design and data analysis.

All authors read and approved the final manuscript.

## Guarantor

Dr. Dong Kyu Moon.

## Provenance and peer review

Not commissioned, externally peer reviewed.

## Appendix A Supplementary data

SCARE Guideline Checklist SCARE Guideline Checklist
